# Relapsing Polychondritis: An Updated Review

**DOI:** 10.3390/biomedicines6030084

**Published:** 2018-08-02

**Authors:** Francesco Borgia, Roberta Giuffrida, Fabrizio Guarneri, Serafinella P. Cannavò

**Affiliations:** Department of Clinical and Experimental Medicine, Section of Dermatology, University of Messina, 98125 Messina, Italy; robgiuffrida@unime.it (R.G.); f.guarneri@tiscali.it (F.G.); cannavop@unime.it (S.P.C.)

**Keywords:** relapsing polychondritis, auricular chondritis, systemic autoimmune disease, cartilage, anti-type II collagen antibodies

## Abstract

Relapsing polychondritis is an immune-mediated systemic disease characterized by recurrent episodes of inflammation of cartilaginous and proteoglycan-rich tissues, resulting in progressive anatomical deformation and functional impairment of the involved structures. Auricular and nasal chondritis and/or polyarthritis represent the most common clinical features, but potentially all types of cartilage may be involved. Because of the pleomorphic nature of the disease, with non-specific symptoms at the onset, the diagnosis of relapsing polychondritis is often delayed. In this review article we provide a comprehensive look into clinical presentation, laboratory and instrumental investigations, diagnostic criteria, and therapeutic options.

## 1. Introduction

Relapsing polychondritis (RP) is an immune-mediated systemic disease characterized by recurrent inflammatory episodes of cartilaginous and proteoglycan-rich tissues, including the elastic cartilage of the ear and nose, the hyaline cartilage of peripheral joints, the fibrocartilage at axial sites and the cartilage of the tracheobronchial tree, which result in progressive anatomical deformation and functional impairment of the involved structures. In over 80% of patients, RP is disclosed by auricular chondritis and polyarthritis, though many organs can be potentially involved. Its onset is often insidious, with acute painful inflammatory crisis followed by spontaneous remission of variable duration. This may render diagnosis very difficult at an early stage, with therapeutic delay and consequent increased risk of permanent or life-threatening sequelae. Association with other autoimmune disorders is found in 30% of all adult RP patients, rheumatoid arthritis (RA) being the most common. In this review article we provide a comprehensive look into clinical presentation, laboratory and instrumental investigations, diagnostic criteria, and therapeutic options, with a focus on the role of biologics in the management of refractory patients.

## 2. History and Epidemiology

In 1923, Jaksch-Wartenhorst described for the first time the disease with the name “polychondropathia” [1]; the term “relapsing polychondritis” was introduced by Pearson et al. in 1960, to underline the peculiar intermittent course observed in 12 patients [2]. In 1976, McAdam et al. proposed the first diagnostic criteria for RP, on the basis of the clinical presentation observed in 159 patients [3]; these criteria were later modified by Damiani and Levine [4] and Michet et al. [5]. RP is considered a rare disease (Orpha code: 728), with a large number of single case reports but few patient series reported in literature. The estimated incidence is 3.5/1,000,000/year [6,7], although a lower figure has been reported in a recent population-based cohort study in the UK [8]. The median age of onset is between the fourth and the fifth decade of life, with most of the patients aged between 44 and 51 years at the time of diagnosis [9]; however, RP can occur at any age. Pediatric RP represents <5% of the reported cases, with age at onset varying from 1.7 months to 17 years; clinical presentation is similar to adult RP [10]. Pregnancy does not influence the course of disease; no neonatal cases have been described until now [11]. RP occurs with similar frequency in both genders, although a slight female preponderance has been reported [12]. It affects all ethnic groups, with variability in clinical presentation between Caucasian and Asian populations [13].

## 3. Pathogenesis

The exact pathogenesis of RP is not yet clearly defined. Genetic studies have identified HLA-DR4 as the major risk allele for RP, while a negative association exists between severity of organ involvement and HLA-DR6 [14]; there is no evidence of familial transmission. RP is considered a complex disorder targeting cartilaginous structures, with involvement of both humoral and cell-mediated immune systems. Circulating autoantibodies against collagens II, IX and XI have been detected in RP patients, suggesting that cartilage-specific autoimmunity may play a crucial role in the pathogenesis of RP [15,16,17]. Type II collagen (CII) accounts for 95% of the total collagen content of the cartilage, and may represent a primary target of autoimmunity. Circulating antibodies against CII have been, in fact, detected in one third of RP patients with active disease, with a positive correlation between serum titers and disease severity [15]. Other known target autoantigens are matrilin-1 and the cartilage oligomeric matrix proteins (COMP). Matrilin-1 is a protein of the intercellular matrix, highly expressed in tracheal, nasal, auricular, and chondro-sternal cartilages but not in normal adult joint cartilage; COMP is predominantly found in the extracellular matrix of cartilage, ligaments, and tendons. In a case report, Saxne and Heinegard evidenced that serum levels of the two cartilage matrix proteins varied inversely during monitoring of an RP patient. Raised matrilin-1 levels were measured in the course of a flare-up, possibly reflecting an increased release from damaged cartilage; COMP serum levels negatively correlated with disease activity, with a reduction in the acute phase and a progressive increase in the course of clinical remission, suggesting that higher levels may reflect tissue restoration and de novo synthesis of cartilage [18]. On the contrary, Kemta Lekpa et al. demonstrated, in 21 RP patients, significantly higher COMP levels during the active phase than in the inactive one [19]. Antibodies to matrilin-1, binding to tracheolaryngeal cartilage in vivo, were identified by Hansson et al. in 13 out of 97 RP patients. Positive titers correlated with respiratory symptoms in 69% of cases [20]. Interestingly, seven out of 97 RP patients showed concomitant presence of anti-matrilin-1 and anti-COMP antibodies, suggesting that immunization against COMP may be a consequence of the matrilin-1 mediated process of cartilage destruction. Experimental studies in murine models confirmed that immunization with extracellular matrix proteins can result in the development of a chondritis closely resembling the clinical picture observed in human RP patients [21,22]. The role of cell-mediated immune response in the pathophysiology of RP is supported by several data. Histopathological examination of affected cartilages revealed inflammatory infiltrate composed by various proportions of T-lymphocytes, mainly CD4 T-cells, macrophages, plasma cells, and immune deposits, restricted to perichondrium at an early stage and, later, spreading to cartilage [23,24]. Chemokines consistent with Th-1 profile, namely, interferon, interleukin (IL)-2, and IL-12, are released during this inflammatory process [25]. Furthermore, T-cell response specific to peptides found in collagen type II or matrilin-1 was found in some patients [26]. With disease progression, high expression of proteolytic enzymes was detected in perichondral cells and chondrocytes. Matrix metalloproteinase (MMP)-8, -9, and elastase were expressed only in the perichondral granulation, whereas MMP-3 and cathepsin K and L were detected in both chondrocytes and granulations. The strong correlation between the number of apoptotic cells and the number of MMP-3-positive and cathepsin K-positive cells suggests that cartilage destruction in RP can be sustained not only by perichondral inflammation, but also by intrinsic factors, such as the aforementioned MMP-3 and cathepsin K and L, strongly expressed in the chondrocytes of deteriorated cartilage and released from the same cells after apoptosis [27,28]. As suggested by Ouchi et al. [27], MMP-3 deteriorates many connective and plasma proteins, such as proteoglycan, various type of collagen (IV, V, VII, IX and denatured type I), laminin, fibronectin, elastin, alpha1-proteinase inhibitor, immunoglobulins, and substance P.

The above mentioned data allows us to hypothesize that yet unknown factors (maybe infectious agents and/or mechanical and chemical aggressions) might cause protein degradation with consequent release of cryptic cartilage antigens. In genetically predisposed subjects, immunization can occur against these autoantigens, in part recognizable in CII, matrilin-1, and COMP. This may perpetuate, via release of pro-inflammatory cytokines and recruitment of infiltrating cells in RP lesions, finally resulting in cartilage destruction mediated by MMP released by chondrocytes undergoing apoptosis [28].

## 4. Clinical Manifestations

Chondritis and polyarthritis are the most common clinical features of RP but, since inflammation of cartilaginous tissue potentially may occur at many anatomical districts, the disease often presents with various combinations of heterogeneous, only apparently unrelated, signs and symptoms. RP can also involve other proteoglycan-rich structures, such as the eye, heart valve, and blood vessels. This makes diagnosis very difficult, especially when auricular and nasal involvement is not yet come into view or when constitutional symptoms, including fever, weight loss, night sweats, fatigue, and lympho-adenomegaly, anticipate the onset of characteristic manifestations [12,14].

### 4.1. Chondritis

Mono- or, more frequently, bilateral auricular chondritis is the most common feature of RP, which is observed in up to 90% of patients during the course of the disease, and is the inaugural symptom in 20% of cases [3,5,29]. The onset is abrupt, with painful red to violaceous erythema and edema confined to the cartilaginous part of the ear, typically sparing the lobe, which lacks cartilage. Acute inflammatory episodes tend to resolve spontaneously within few days or weeks, with recurrence at variable intervals. As a long-term consequence of repeated flares, the cartilage matrix is severely damaged and replaced by fibrous connective tissue. The ear progressively loses its normal morphology, appearing nodular or verrucous, floppy or hardened for calcification. In a small percentage of patients, the deformity of the pinna resembles the “cauliflower ear” of professional boxers [29]. Hearing loss, which can be conductive or sensorineural, was demonstrated in as many as 46% of patients with RP, and vestibular dysfunction was documented in 6%. Conductive hearing loss was reported secondary to auricular cartilage collapse, edema of the canal, closure of the external auditory meatus leading to serous otitis media, and stapedial foot plate fixation [30,31]; inflammation of the vestibular structures or vasculitis of the internal auditory artery may cause sensorineural hearing loss [12,30]. Otitis externa, chronic myringitis, and tinnitus were reported as well [32].

Nasal chondritis is present at the time of diagnosis in 24% of patients, and develops subsequently in 53% of cases [29]. The inflammatory process involves the nasal bridge, with acute redness, tenderness, and pain, usually less marked than in the ears. It can be occasionally accompanied by epistaxis [30]. Progressive destruction of nasal cartilage leads to the characteristic flattening of the nasal bridge, finally resulting in the painless, irreversible “saddle nose” deformity, more frequently observed in female patients and those under 50 years [7,12].

Laryngotracheobronchial involvement is seen at presentation in only 10% of cases, but it eventually develops in half of all patients, more commonly in females [3,33]. When inflammation is limited to the larynx, initial symptoms include pain and tenderness over the thyroid cartilage and trachea, leading to laryngomalacia or permanent stenosis with hoarseness of voice, non-productive cough, dyspnea, stridor, and wheezing, that may require emergency tracheotomy as a temporary or permanent measure [3,7,34]. Tracheobronchial involvement has a poor prognosis, being the major cause of morbidity and mortality [3]. The thickening of the tracheal wall with destruction of the cartilaginous rings is characteristic. Tracheomalacia may be observed, with resultant airway collapse [35]. Fixed narrowing and stenosis may develop from granulation tissue and peribronchial fibrosis. Strictures in the airways from chronic inflammation, fixed or dynamic, might generate subglottic inflammation, tracheal collapse, and secondary pulmonary infections [36]. Airway intervention is frequently required, by means of balloon dilatation, stent placement, tracheotomy, or a combination of the above mentioned techniques [35]. The involvement of costal cartilages occurs in 35% of patients, but it is rare at the moment of diagnosis. It causes chest wall pain or swelling of the involved cartilages.

### 4.2. Arthropathy

This is the second most frequent symptom in RP and appears in approximately 50–85% of patients during the disease, but in only 33% of these it is an initial feature [12,37]. The main pattern of joint involvement is acute asymmetric intermittent polyarthritis or oligoarthritis affecting metacarpophalangeal joints, proximal interphalangeal joints, knees, and, less commonly, ankle, wrists, metatarsophalangeal joints, and elbows [37,38]. Usually, no erosions or deformities are observed, although a few cases with joint destruction have been reported [39]. Axial involvement has rarely been described, and tendinopathy and tenosynovitis are reported only in few cases [40].

### 4.3. Ocular Manifestations

Ocular manifestations are present in 50–60% of cases of RP but are rarely inaugural. They are usually mild and consist, in order of frequency, of episcleritis (unilateral or bilateral), scleritis (often anterior, lingering or relapsing, exceptionally necrotizing), and conjunctivitis [3,29]. Less frequently, RP can cause iritis, retinopathy, muscle paresis, anterior uveitis, optic neuritis, orbital inflammation, keratoconjunctivitis sicca, peripheral keratitis, retinal vasculitis, occlusion of the retinal arteries or veins, ischemic optic neuritis, and cataract related to the disease or steroid-induced [41,42]. Sometimes eyes are the initial site of involvement and, since most patients with ocular inflammation tend to develop multiple systemic manifestations, this may be regarded as a marker of severity.

### 4.4. Neurologic Manifestations

These affect 3% of patients with RP, most commonly with involvement of the V and VII cranial nerves [12]. Symptoms are often related to a concomitant vasculitis of the central or peripheral nervous system [43]. Clinical manifestations include headache, meningitis, limbic encephalitis, cerebral infarction, hemiplegia, ataxia, seizures, confusion, psychosis, and dementia [44,45,46,47,48,49]. Regarding cognitive dysfunction, Ellis et al. have suggested the existence of two distinct phenotypes. The first is a fulminant, multisystem presentation with sub-acute cognitive decline mimicking central nervous system vasculitis, whereas the other is an insidious cognitive decline without associated constitutional or systemic symptoms [50].

### 4.5. Renal Manifestations

Renal complications of RP are rare. About 22% of patients with RP develop some type of renal lesion, with microhematuria and/or proteinuria, but biopsy-proven nephropathy has been reported in less than 10% of patients [51,52]. Renal involvement is associated to poor prognosis, with a 10-year survival rate of 10% [52]. Renal pathology may manifest as mesangial expansion, IgA nephropathy, tubulointerstitial nephritis, segmental necrotizing crescentic glomerulonephritis, and membranous nephropathy [52,53,54]. According to previous reports, immunofluorescence microscopy of kidney biopsy specimens inconstantly shows IgA, IgG, IgM, and complement deposits in the basement membrane, capillary walls, and mesangium, suggesting that immune complexes may play a role in the pathogenesis of the glomerular lesions of RP [51,52].

### 4.6. Dermatological Manifestations

Cutaneous features have been reported in 17% to 37% of patients with RP, usually occurring simultaneously with or after chondritis [3,55]. The most frequent skin manifestations are aphthosis, nodules localized to limbs, raised purpura and papules, livedo, and distal ulcerations and necrosis related to concomitant vasculitis, usually considered as poorly specific. Recently, Tronquoy et al. described the occurrence, in ten patients, of tense urticarial papules, frequently with annular shape, predominantly located on the upper part of the trunk, shoulders, neck, and less frequently on the proximal part of the limbs. Histologic examination consistently showed a lymphocytic vasculitis with no leukocytoclastic vasculitis. Interestingly, in contrast with previous reports, skin lesions occurred before the diagnosis of RP in seven of 10 cases, with a mean delay of RP of 23 months [56]. The concomitant presence of oral and genital aphtous ulcerations and chondritis has been first described by Firestein, who coined the acronym MAGIC (mouth and genital ulcers with inflamed cartilage) to indicate a syndrome which includes diagnostic criteria of RP and Behçet’s disease (BD). Some Authors suggested the existence of a common autoimmune pathogenic mechanism [57], but the pathogenic association between these three diseases is still unclear.

### 4.7. Cardiovascular Manifestations

Cardiovascular complications are diagnosed in about 25% of patients with RP, especially in males, and represent the second most frequent cause of mortality [5]. The clinical spectrum includes valvular heart disease, aortic aneurysm, aortic dissection, myocarditis, pericarditis, atrioventricular block, and systemic vasculitis. Heart valve incompetence occurs in about 10% of patients. Aortic regurgitation is reported in 4% to 6% of patients, secondary to dilation of the aortic ring combined with ectasia of the origin of the aorta. Mitral regurgitation develops in 2% to 4% of patients [3,5,58]. Due to the silent, slow development of valvular damage, RP patients need to be strictly monitored with periodic echocardiographic evaluation. Aortic aneurysms are not rare; they may be multiple and located in all parts of the aorta, even resulting in fatal rupture in asymptomatic patients [59]. Other manifestations include obstructive lesions and silent myocardial infarction [5,12,29]. Vasculitis of any vessel may be seen, with a clinical spectrum ranging from cutaneous leukocytoclastic vasculitis to large vessel vasculitis, mimicking Takayasu’s arteritis, Churg-Strauss syndrome, polyarteritis nodosa, and granulomatosis with polyangiitis [60,61,62,63].

### 4.8. Associated Disorders

A long list of diseases occurring in association with RP is reported, with an estimated incidence of 30% of cases [4]. It includes autoimmune disorders (systemic lupus erythematosus, systemic sclerosis, mixed connective tissue disease, Sjögren’s syndrome, dermatomyositis), rheumatological diseases (spondyloarthropathy and, with high frequency, rheumatoid arthritis), and vasculitis [64]. An increasing number of RP cases have been described as being linked to malignancies, particularly myelodysplastic syndrome (MDS) and, albeit less frequently, solid tumors (bladder, breast, lung, colon, pancreas) or other hematological malignancies (lymphoma). The association of RP with MDS has been well reported in literature, with up to 27% of RP patients having concomitant MDS [55,65]. Sweet’s syndrome and RP are very rarely found together in the same patient. This dual occurrence is more commonly found in cancer patients with associated hematological malignancies [66].

## 5. Diagnosis and Prognosis

The diagnosis of RP is a real challenge for clinicians, because of the pleomorphic nature and insidious onset of the disease. Quite simple when the typical involvement of ear and nose cartilage is present, diagnosis is often missed, especially at early stages, when nonspecific, sporadic, remittent inflammatory episodes happen with no apparent link, justifying the mean diagnostic delay of 2.9 years [12]. In children, the remitting-relapsing natural history of RP and the poor knowledge of this condition by pediatricians may account for the mean five-year delay [10]. The diagnosis of RP is still based on clinical grounds (Table 1), because there are no specific laboratory tests, histological patterns, or imaging. According to McAdam et al., the diagnosis of RP can be made if three or more of the six clinical features (auricular chondritis, nonerosive inflammatory polyarthritis, nasal chondritis, ocular inflammation, respiratory tract chondritis, audiovestibular damage) are present, requiring no histologic confirmation [3]. These criteria were later modified by Damiani and Levine, who expanded the spectrum of diagnostic criteria, adding the presence of at least one McAdam criterion and positive histologic confirmation, or two McAdam criteria and positive response to administration of corticosteroids or dapsone [4]. Another variant of McAdam criteria was proposed by Michet et al. in 1986 [5]; according to the latter, the diagnosis of RP requires a confirmed inflammation in two of three auricular, nasal or laryngotracheal cartilages, or else a proven inflammation in one of the above cartilages and two other minor criteria among hearing loss, ocular inflammation, vestibulary disfunction, seronegative arthritis.

Laboratory findings may be suggestive of inflammation and sometimes organ damage, but there are not specific laboratory tests for the diagnosis of RP. The levels of C-reactive protein and erythrocyte sedimentation rate are usually raised during the inflammatory crisis, but findings of normal values during the remission phase do not allow exclusion of the diagnosis. Blood exams may reveal anemia, leukocytosis, thrombocytosis, polyclonal hyper-gammaglobulinemia; serum creatinine and urinalysis are useful to detect renal function impairment [29]. Other laboratory investigations, such as rheumatoid factor, antinuclear antibody (ANA), anti-phospholipid antibodies, and complement levels, can be useful to evidence the presence of concomitant diseases [14]. The prevalence of ANA observed in RP is low, and the finding of a significant titre of ANA in a patient with RP strongly suggests the presence of an associated disorder [67]. Anti-neutrophil cytoplasmic antibodies (ANCA) can be present in up to 25% of patients with RP and concomitant ANCA-associated vasculitis [68], but they may also be an isolated finding in RP or precede the onset of vasculitis [53,69].

Identification of anti-type II collagen antibodies in the acute phase of RP represents another low specificity-finding [70], whereas their serum levels seem to correlate with disease severity [15].

A positive correlation between raised serum levels of anti-matrilin-1 antibodies and the presence of respiratory symptoms has been reported by Hansson et al. [20]. Even COMP levels may be high in the active phase of the disease [19]. Histology does not add weighty diagnostic information and biopsy of the cartilage is not routinely performed, with the exception of atypical clinical presentation. The detection of basophilic staining of the cartilage matrix, perichondral round-cell infiltration or cartilage destruction with fibrous replacement can be of diagnostic value [12]. X-ray examination may be helpful to evidence calcification of auricolar, nasal, tracheal, and articular cartilage, which may be found in chronic RP. The sensitivity of conventional chest radiography is not sufficient for accurate visualization of RP. Early identification of airway involvement is mandatory for prompt diagnosis and aggressive treatment, which may prevent a potentially fatal outcome. The CT findings in patients with RP consisted mainly of airway wall thickening, airway stenosis, airway malacia, airway wall calcification, and air trapping [71]. Expiratory CT abnormalities are present in the majority of RP patients, yet only half of patients demonstrated abnormalities on routine inspiratory CT scans. According to Lee et al., dynamic expiratory CT should be considered a routine diagnostic component if there is clinical suspicion of airway involvement [72]. However, stenosis may not improve after suppression of inflammation, making CT assessment following therapy difficult in some cases. MRI plays a major role in the diagnosis of RP by demonstrating a unique pattern of inflammation and enhancement, that preferentially involves the perichondrium and chondroepiphysis, even in the early phases of the disease. Recently, encouraging results have been reported with fluorodeoxyglucose (FDG)-PET/CT, that promises to be a useful tool for both diagnosis and evaluation of disease activity [73]. Bronchoscopy is accompanied by an elevated risk of exacerbation of airway inflammation and may induce potentially fatal respiratory distress, so its use is limited to selected cases [35]. Echocardiography is used to assess cardiac valves and aortic root in RP patients with suspected cardiovascular involvement. A recent report highlights the utility of PET-CT in detecting aortic root dilatation secondary to aortitis with critical coronary artery stenosis [74]. Bone scintigraphy using 99mTc-methylene diphosphonate and gallium-67 citrate has been reported to demonstrate inflammatory cartilage and joints and may be employed for evaluating inflammatory activity and monitoring treatment responses in RP patients [75,76].

A specific disease activity score, called the Relapsing Polychondritis Disease Activity Index (RPDAI), has been developed by a panel of RP experts in 2012 in order to standardize the evaluation of patients and to monitor response to treatment [77]. The RPDAI score is made up of 27 items with individual weights ranging from 1 to 24 and a maximum theoretical RPDAI score of 265, taking into account disease manifestations in a 28-day period (online scoring at www.RPDAI.org). Preliminary results of RPDAI application seem to confirm the potential utility of this tool in monitoring the clinical course of the disease [78].

Survival rates have increased from 70% after five years to 94% after eight years, and 91% after 10 years in a recent large single-center study [40]. These improvements can be explained in the light of improved diagnostic ability, with detection of the disease at an early stage, and availability of new drugs. Causes of death in RP include disease-dependent direct organic damage, concomitant diseases, and infections, which may be an undesired consequence of immunosuppressive treatments [79].

## 6. Therapy

There are no evidence-based guidelines for the treatment of RP. Available therapeutic options are summarized in Table 2. The goal of therapy is the control of the inflammatory crisis and the long-term suppression of the immune-mediated pathogenetic mechanisms. The ideal therapy should allow the achievement of rapid relief of symptoms and the prevention of multi-organ effects on cartilaginous structures, with the fewest side effects, taking into consideration the need for chronic administration. Non-steroidal anti-inflammatory drugs (NSAIDs) may be used for pain control and inflammation in non-severe forms of RP, characterized by involvement of nose, external ear, or joints only [80,81]. Mild manifestations can be also managed with dapsone (50–100 mg, once daily; maximum dose of 200 mg, once daily) or colchicine (0.6 mg 2–4 times daily) [82,83,84]. In the case of NSAIDs resistance or in severe forms including ocular, laryngotracheal, or cardiac involvement, systemic vasculitis and severe polychondritis, systemic corticosteroids are considered the treatment of choice [85,86]. Oral prednisone is usually started with a dose ranging from 0.25 to 1 mg/kg daily, reducing the dose, if possible, during the course of the disease [80,86]. If a rapid effect is necessary, intravenous pulse methylprednisolone (500–1000 mg/day) can be helpful [86].

Continued steroid therapy is often recommended in long-term follow-up to prevent relapses, but it does not modify the progression of the disease. For this reason, several other drugs such as cyclophosphamide (1 mg/kg/day for two weeks, increasing the dose by 25 mg every two weeks), azathioprine (2 mg/kg/day), cyclosporine (5 mg/kg/day), and methotrexate (15–25 mg/week orally or subcutaneously) have been used, alone or in association with systemic corticosteroids, as second line options in case of organ or life threatening disease [8,12,40,70,81,87,88]. Their use is also indicated in corticosteroid-intolerant or corticosteroid-dependent patients or in cases of lack of response to corticosteroids or necessity of corticosteroid-sparing therapy [86,88,101]. In recent years, the arrival of biologics has opened new perspectives for patients resistant to classical immunosuppressive treatments. In 2012, Kempta-Lepka et al. reviewed the literature concerning treatment of 62 patients affected by active RP with tumor factor necrosis (TNF) blockers (infliximab, etanercept and adalimumab), rituximab (monoclonal antibody directed against the CD20 antigen on B lymphocytes), anakinra (Interleukin-1 receptor antagonist), tocilizumab (humanized anti-human interleukin-6 receptor monoclonal antibody), and abatacept (soluble fusion protein that inhibits T cell activation by binding to CD80 and CD86) [89]. Biologics proved effective in 28 patients, partially effective in six patients, and not effective in 28 patients. In particular, the use of infliximab (3–10 mg/kg every 6–8 weeks) [89] induced complete or partial remission of inflammatory signs and complications in 18 out of 31 RP patients treated. The efficacy of etanercept (50 mg/week) and adalimumab (40 mg/2 weeks) was tested respectively in eight and four patients; the first was effective in five patients, while the last in two. The efficacy of rituximab is still debated, with some authors who reported improvement [91,92] of the clinical course of the disease and others who found the drug ineffective [86]. Anakinra (100 mg/day) has been used in a small amount of patients, particularly after a lack of efficacy of other biologics [86,93,94]. Promising results have been documented with tocilizumab (8 mg/kg/month), with serial MRI imaging showing response to treatment in progressive RP with visceral involvement [90]. Abatacept was used successfully (750 mg/month) in three patients with RP by Moulis et al. [95,96]. Peng et al. tested abatacept in four patients at the dose of 125 mg/week for 24 weeks. In two cases the treatment was discontinued early for worsening of organ disease. However, chondritis improved in three out of four cases [97]. In general, data show that biologics may help and lead to improvements in cases of RP refractory to conventional therapy. However, randomized controlled trials, although not easy to perform due to the rarity of the disease, are needed to assure efficacy and safety of biologics in RP.

Limited experiences with other treatments, such as 6-mercaptopurine, plasmapheresis, anti-CD4 monoclonal antibody, penicillamine, minocycline, high-dose intravenous immunoglobulins and leflunomide, have been reported in literature, with mixed results [86,98,99,100].

In selected cases, complicated by severe bronchial stenosis or intractable cardiac failure because of valve regurgitation, and in the event of aortic aneurysms, surgical or interventional procedures could be necessary [80].

## 7. Conclusions

RP is a rare and potentially fatal multisystemic autoimmune disease of unknown etiology, affecting primarily cartilagineous and proteoglycan-rich structures. Patients present with a wide spectrum of clinical manifestations that allow diagnosis to be made. To date, therapy of RP is still empiric, due to the lack of standardized guidelines on treatment, and is defined on the basis of disease activity and severity of organ involvement. Mild forms are treated with NSAIDs, colchicine, dapsone, and low-dose corticosteroids. Life-threatening or organ-threatening complications require high-dose corticosteroids and immunosuppressants. Biologics are a new weapon in the war against autoimmune diseases, such as RP. However, the number of patients treated are still limited, and the majority of studies is heterogeneous and with different outcomes. Moreover, little laboratory and clinical research has been conducted on human RP recently, as demonstrated by the limited number of papers published in the last years. For these reasons, clinical trials are needed and biologics should be used after the failure of conventional immunosuppressive treatments.

## Figures and Tables

**Table 1:** Diagnostic criteria of relapsing polychondritis, according to different authors.

Authors, Year and Reference	Suggested Criteria
Mc Adam et al. 1976 [3]	At least three clinical features among auricular chondritis, nonerosive inflammatory polyarthritis, nasal chondritis, ocular inflammation, respiratory tract chondritis, audiovestibular damage; histologic confirmation not required
Damiani and Levine 1979 [4]	At least one of the six clinical features suggested by Mc Adam et al. [3] plus histological confirmation *or* two of the six clinical features suggested by Mc Adam et al. [3] plus positive response to administration of corticosteroids or dapsone
Michet et al. 1986 [5]	Confirmed inflammation in two of three cartilages among auricular, nasal or laryngotracheal *or* proven inflammation in one of the above cartilages plus two other minor criteria among hearing loss, ocular inflammation, vestibulary disfunction, seronegative arthritis

**Table 2:** Therapeutic options for relapsing polychondritis.

Indications	Treatment	References	Notes
Control of pain and inflammation in non severe forms	Non-steroidal anti-inflammatory drugs (NSAIDs)	[80,81]	
Mild manifestations	Dapsone, Colchicine	[82,83,84]	
NSAIDs resistance Severe forms including ocular, laryngotracheal or cardiac involvement, systemic vasculitis and severe polychondritis	Systemic corticosteroids	[85,86]	Oral prednisone is commonly used; intravenous pulse methylprednisolone for rapid effect. Continued steroid therapy is often recommended in long-term follow-up to prevent relapses, but does not modify disease progression.
Second line options in organ- or life-threatening disease Corticosteroid-intolerant or corticosteroid-dependent patients Lack of response to corticosteroids Need for corticosteroid-sparing therapy	Cyclophosphamide, Azathioprine, Cyclosporine, Methotrexate (alone or in association with systemic corticosteroids)	[8,12,40,70,81,86,87,88]	
Resistance to classical immunosuppressive treatments	Biologics (Infliximab, Etanercept, Adalimumab, Rituximab, Anakinra, Tocilizumab, Abatacept)	[86,89,90,91,92,93,94,95,96,97]	Limited clinical experience (62 patients in total, no randomized controlled trials). Overall, effective in 28 patients, partially effective in 6 patients, and not effective in 28 patients.
No specific indication	Other treatments (6-mercaptopurine, plasmapheresis, anti-CD4 monoclonal antibody, penicillamine, minocycline, high-dose intravenous immunoglobulins, leflunomide)	[86,98,99,100]	Limited or anecdotal experience, with mixed results
Selected cases, complicated by severe bronchial stenosis or intractable cardiac failure because of valve regurgitation, and in the event of aortic aneurysms	Surgical or interventional procedures	[80]	
